# Femoral Neck Fracture Management in Elderly Patients: Surgeons’ Practice Through a Registry Analysis

**DOI:** 10.3390/life15101503

**Published:** 2025-09-24

**Authors:** Corrado Ciatti, Virginia Masoni, Fabrizio Rivera, Luca Andriollo, Barbara Bordini, Fabrizio Quattrini

**Affiliations:** 1 Department of Medicine and Surgery, University of Parma, 43126 Parma, Italy; 2 Department of Orthopedics and Traumatology, Guglielmo da Saliceto Hospital, Via Taverna 49, 29121 Piacenza, Italy; 3 Department of Orthopedics and Traumatology, University of Turin, Via Zuretti, 29, 10126 Turin, Italy; virginia.masoni@unito.it; 4 Department of Orthopedics and Traumatology, Ospedale SS Annunziata, ASL CN1, Via Ospedali, 9, 12038 Savigliano, Italy; rivgio@libero.it; 5 Ortopedia e Traumatologia, Fondazione Poliambulanza Istituto Ospedaliero, 25124 Brescia, Italy; luca.andriollo01@icatt.it; 6 Dipartimento di Ortopedia, Università Cattolica del Sacro Cuore, 00168 Rome, Italy; 7 Artificial Intelligence Center, Alma Mater Europaea University, 1030 Vienna, Austria; 8 Laboratory of Medical Technology, IRCCS Istituto Ortopedico Rizzoli, 40136 Bologna, Italy; barbara.bordini@ior.it

**Keywords:** femoral neck fractures, elderly patients, registry analysis, hemiarthroplasty, hip replacement

## Abstract

Background: Femoral neck fractures (FNFs) in elderly patients are a growing concern given increased life expectancy and functional demands. Hemiarthroplasty is the standard treatment, but optimal fixation, approach, and perioperative management remain debated. This study aims to describe implant characteristics, perioperative details, survival, and complications of hemiarthroplasty in patients aged ≥75 years. Methods: A descriptive retrospective analysis was performed using the Emilia Romagna arthroplasty registry (RIPO). All patients ≥ 75 years who underwent hemiarthroplasty for FNFs between 2000 and 2021 were included. Data on demographics, implant fixation, surgical approach, complications, and revisions were analyzed. Implant survival was assessed with Kaplan–Meier analysis. Results: A total of 43,657 procedures were identified; the mean age was 85.5 years, and 73.7% were female. Cemented stems were used in ~76% of cases. The lateral approach was most common (52.6%), followed by posterolateral (43.7%) and anterior (2.9%). Overall revision rate was <2% (853 cases). Dislocation was the leading cause of failure (46.9%), followed by periprosthetic fracture, acetabular wear, aseptic loosening, and infection. Heparins were used for thromboprophylaxis in >93% of cases. Ninety-day mortality reached 13.9%. Conclusions: In this large registry study, cemented stems and the lateral approach were predominant. Despite low revision rates, dislocation remained the main cause of failure. High perioperative mortality highlights the vulnerability of this population and the importance of multidisciplinary care. Future high-quality studies, as prospective studies, will be necessary to determine the optimal solutions in this frail elderly population.

## 1. Introduction

Hip fractures, particularly those involving the femoral neck, have become an increasingly relevant clinical and social concern over the past decades [1,2,3]. The rising incidence of these injuries is strongly related to the progressive aging of the population, with a steady increase in life expectancy worldwide [3,4]. As older adults live longer, their functional demands remain high, making the restoration of mobility and independence a key therapeutic goal after hip fracture [4,5,6,7].

In patients 75 years of age and over, femoral neck fractures (FNFs) are often managed with hemiarthroplasty (HA), which allows for earlier mobilization and reduces the risk of complications associated with prolonged immobilization [3,4,5,7,8,9]. Nevertheless, this population carries a substantial perioperative risk, and mortality remains a major concern [8,10,11,12]. Indeed, the estimated one-year mortality rate was 26.8% after head/neck fracture, as reported by Walter et al. [12]. Therefore, careful perioperative management and the choice of surgical technique are crucial to improving outcomes [8,13,14,15,16].

Among the technical considerations, the decision to use a cemented or uncemented stem continues to generate debate [8,9,11,14,16]. Cementation is widely adopted in elderly patients since it provides immediate stability and it reduces the risk of periprosthetic fracture; however, it also introduces specific hazards, such as bone cement implantation syndrome (BCIS), which must be weighed against potential benefits [8,10,11,14,16,17,18,19]. Cementing techniques have been improved, with, for example, the use of vacuum mixing and epinephrine-soaked sponges to remove the blood from the femoral canal [17]. Furthermore, the materials, such as the cement, have been modified. In this context the polymethylmethacrylate (PMMA) was one of the most widely used biomaterials; however, concerns regarding the risk of aseptic loosening of bone-cement bonding have been raised [20,21,22].

As reported by Karpiński et al., to enhance its mechanical properties and to reduce this risk of cement loosening due to the surgical field contamination by blood, saline, and other lavage solutions, additives such as tricalcium phosphate, glassy carbon, or hydroxyapatite have been added and are well-described in the current literature. These particles have been added in different formulations and mixtures [20,21,22].

Moreover, the choice of surgical approach plays an essential role, influencing not only intraoperative complications but also the postoperative recovery, the stability, and the long-term functional outcomes [13,15,23].

Taken together, these factors highlight the complexity of managing FNFs in elderly patients [1,2,7]. The optimization of surgical strategies, including the use of cemented stems and the selection of the most appropriate surgical access, remains paramount [8,10,11,14,16,17,18,19].

The aim of this study is to describe demographics and surgical details along with survival and complication rates of HA implants used in the treatment of FNFs in patients aged 75 years and older through a registry analysis. Particular attention is directed to reporting which implant characteristics and surgical strategies, including stem fixation and surgical approach, have been adopted. By analyzing these factors, this study aims to provide insight into optimizing implant selection and perioperative management strategy in this fragile yet functionally demanding patient population.

## 2. Materials and Methods

This retrospective observational study was conducted using data from the regional arthroplasty register of Emilia-Romagna, known as the Registro dell’Implantologia Protesica Ortopedica (RIPO, Bologna, Italy) [24,25]. Emilia-Romagna is a region in northern Italy with approximately 4 million inhabitants, featuring 69 active orthopedic units in 63 hospitals, both public and private [25,26,27]. The RIPO was established in 1990 at the Istituto Ortopedico Rizzoli in Bologna [25,26,27,28]. It started 1 January 2000, and it collects data on all hip, knee, and shoulder arthroplasties performed throughout the region [25,26,27,28]. Data collection is manually gathered on a paper form and transmitted to the RIPO, which verifies the data and enters them into the registry website (https://ripo.cineca.it (accessed on 17 February 2025)) [25,26,27,28]. Each year, a public report is published in both English and Italian [25,26,27,28].

As of 31 December 2023, the Register had collected data on nearly 165,000 total hip arthroplasties (THA), 54,000 hemiarthroplasties, 22,100 revisions, 148,000 knee replacements, 10,500 knee revisions, and 13,000 shoulder prostheses [28]. It covers more than 95% of surgical procedures performed in the region, ensuring the accuracy of the analysis executed [25,26,27,28]. It contributes to the Registro Italiano Artroprotesi (RIA), which is the national Italian arthroplasty registry [29].

As a general rule, the descriptive analyses are performed on all cases [25,28]. In contrast, survival analyses are performed only on patients living in the Emilia-Romagna region to avoid bias resulting from the ‘loss’ of non-resident patients, since it is not always possible to know the rationale for revision if they are carried out outside the region [25,28]. For this reason, only patients living in Emilia Romagna were included in the present analysis to ensure complete follow-up [25].

Data extraction from the RIPO database was performed on 27 September 2024.

**Figure 1:**
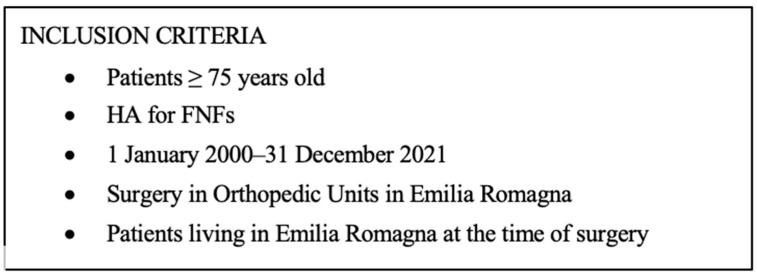
Inclusion criteria. All criteria are required to be included.

The study cohort included all patients aged 75 years and older who underwent HA for FNFs between 1 January 2000, and 31 December 2021. No restrictions were applied with respect to fracture classification. No exclusion criteria were applied concerning the ASA score, the functional status, the living situation, and the pre-fracture function of the patient. As already mentioned, patients not living in Emilia Romagna were excluded to minimize the risk of incomplete follow-up (Figure 1).

Demographic data, implant characteristics, surgical approaches, and fixation methods were extracted from the registry. Failures and revisions were recorded, and implant survival was analyzed.

The causes of failures were reported.

Concerning revisions, three big groups, such as the conversion to THA, the sole revision of HA, or the removal of the prosthetic materials, were firstly described. Then they were analyzed as subtypes in detail.

Moreover, the distribution of the causes of failures and revision types according to the timeline was outlined to understand if there were specific patterns. Even in this analysis, subtypes of revisions were considered.

Ethical approval was not required, as the registry collects data as part of standard clinical practice, and all information was analyzed in a de-identified and anonymous format to protect patient confidentiality.

Statistical Analysis

Patient demographics and implant characteristics were described as mean values with standard deviations or medians with interquartile ranges for continuous variables and as absolute frequencies with percentages for categorical variables. Causes of failures and revision procedures were reported as percentages and rates.

When considering body mass index (BMI), the WHO criteria were followed, with underweight defined as BMI < 18.5, overweight with BMI ≥ 25, and obese with BMI ≥ 30 [30].

Implant survival was evaluated using Kaplan–Meier analysis, with revision of any component defined as the endpoint, with numbers at risk. Patients without revision were censored at the time of death or at the last available follow-up (31 December 2021). In addition, implant survival with the competing risk of death was evaluated using Kaplan-Meier analysis, with revision of any component defined as the endpoint, along with numbers at risk.

The statistical analysis was performed using R Core Team 4.3.2 (2023). R: A Language and Environment for Statistical Computing. R Foundation for Statistical Computing, Vienna, Austria (https://www.R-project.org/ (accessed on 27 September 2024)).

## 3. Results

Between 2000 and 2021, a total of 43,657 HAs performed for FNFs in patients aged 75 years and older were registered in the RIPO database and included in the present analysis. The mean age at the time of surgery was 85.5 years (SD 5.5). Most patients were women (73.7%). The demographic details are reported in Table 1.

Mortality was high in this fragile population: 13.9% of patients died within 90 days from surgery, while 74.0% died after 90 days.

93.4% of patients were administered heparins for thromboprophylaxis in the postoperative period. Concerning blood transfusions, only 38.0% did not require them. Perioperative details are reported in Table 2.

Cemented stems were used in most cases at 75.9%, with 71.2% of cases not having antibiotics and 4.7% adding antibiotics, respectively. On the contrary, uncemented stems accounted for approximately one-quarter of the cohort. 98.9% of cups were bipolar. The femoral head components were almost exclusively ≤28 mm (99.1%) and metallic (98.5%).

Regarding surgical approach, the direct lateral approach was the most frequently adopted (52.6%), followed by the posterolateral (43.7%) and the anterior approach (2.9%). Implant details are reported in Table 3.

A total of 853 failures and revisions were identified during the study period, corresponding to an overall failure of less than 2%. The leading cause of revision was dislocation, which accounted for nearly half of failures, followed by periprosthetic fracture, acetabular wear, and both aseptic and septic loosening, respectively. The complete distribution of failure causes is provided in Table 4.

As for the revision procedures performed, conversion to total hip arthroplasty with acetabular cup positioning accounted for most revision procedures. Isolated HA stem, head, or neck revisions were rare (Table 5 and Table 6).

**Figure 2:**
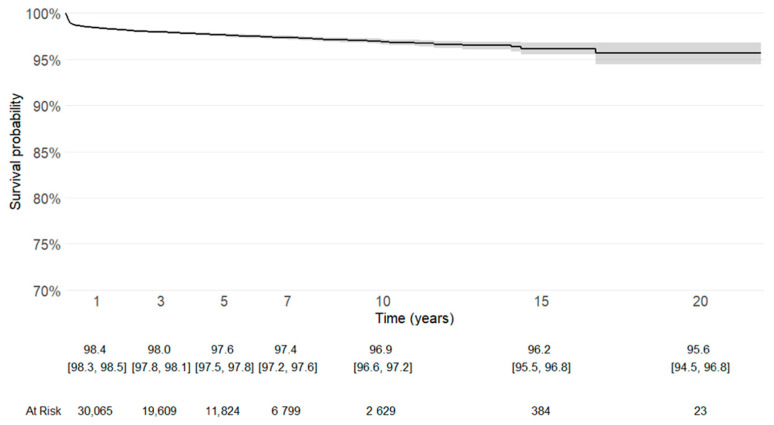
Kaplan–Meier survival curve with revision for any cause or removal of the prosthetic material as the endpoint. Patients without revision were censored at the date of death or at last follow-up (31 December 2021). (95% confidence interval [CI], 94.5–96.8%).

Kaplan–Meier survivorship analysis, with revision for any cause or removal of the prosthetic material as the endpoint, confirmed satisfactory long-term implant survival (Figure 2).

**Figure 3:**
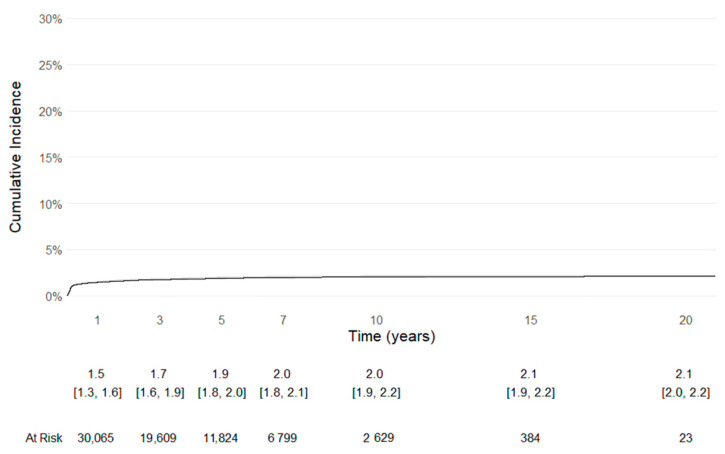
Kaplan-Meier survival curve with the competing risk of death, with revision of any component defined as the endpoint.

Even Kaplan–Meier survivorship analysis, with the competing risk of death, with revision of any component defined as the endpoint, confirmed the longevity of the implants (Figure 3).

### Distribution of the Causes of Failure and Revision Types According to the Timeline

**Figure 4:**
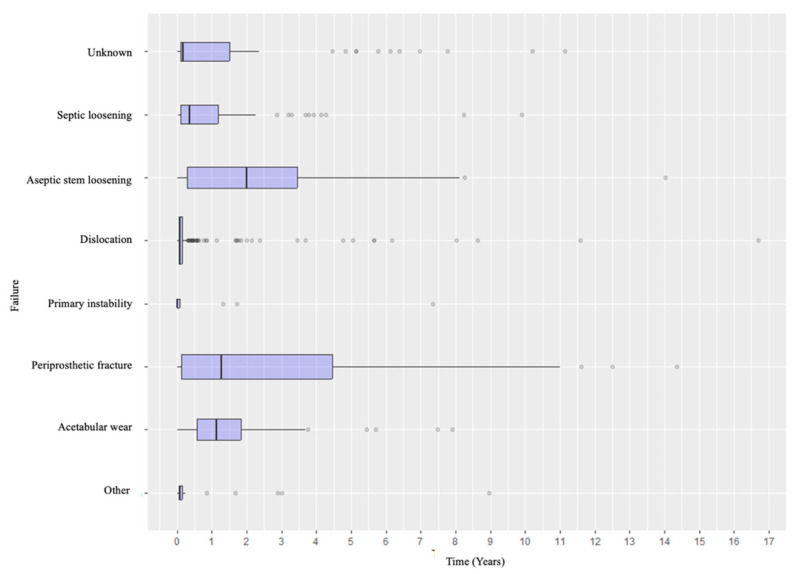
Distribution of the causes of failure according to the timeline.

**Figure 5:**
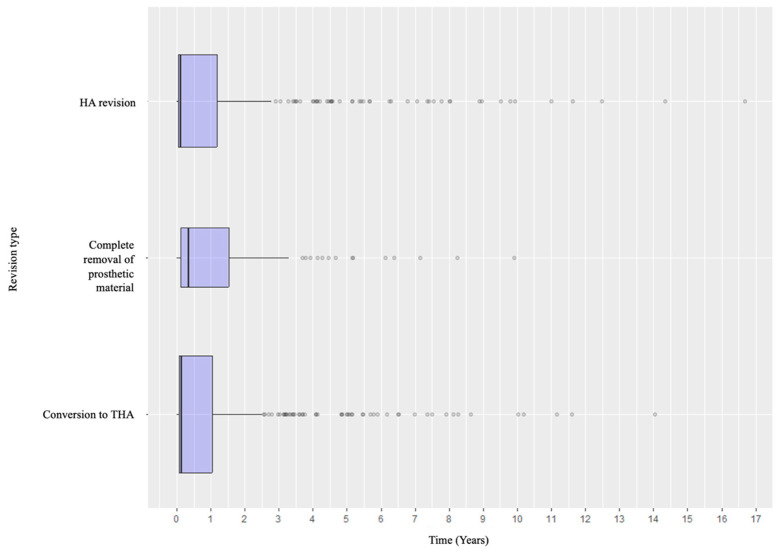
Distribution of the revision types according to the timeline.

**Figure 6:**
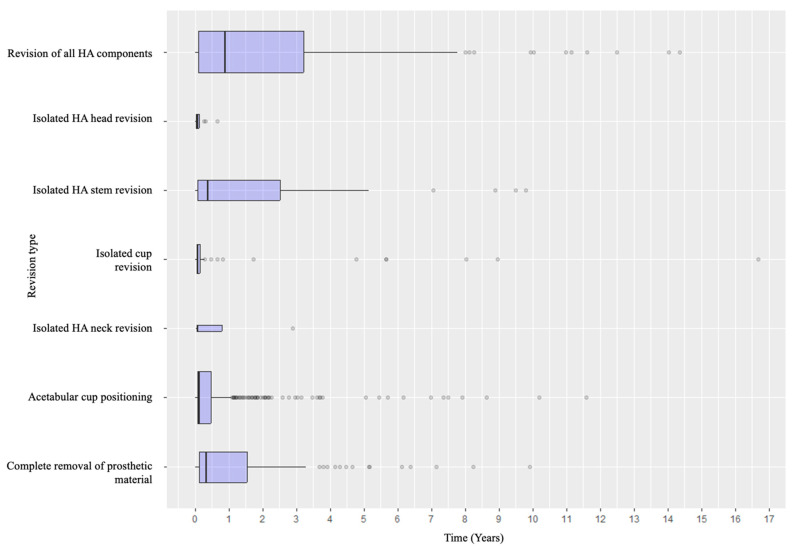
Distribution of the detailed revision types according to the timeline.

Specifically, when analyzing the timing of failure presentation, different patterns emerged. Dislocation was the most common early complication, with most events occurring within the early postoperative years. In contrast, aseptic and septic loosening, as well as acetabular wear, tended to appear later. Periprosthetic fractures tended to occur in the early intraoperative or perioperative period and in the medium- to long-term follow-up (Figure 4). Revision types also varied according to timing: early revisions were predominantly due to dislocation and managed with acetabular procedures, while late revisions more frequently involved loosening or wear and thus required conversion to total hip arthroplasty or complete removal of the prosthetic material (Figure 5 and Figure 6).

## 4. Discussion

The management of FNFs in elderly patients is a multifactorial issue that involves not only surgical technique but also perioperative care, patient comorbidities, and rehabilitation strategies. The present study, which describes more than 43,000 cases recorded in the RIPO registry, provides a unique descriptive overview of implant characteristics and perioperative details, survival, and complications of hemiarthroplasty in the treatment of femoral neck fractures in patients aged 75 years and older.

### 4.1. Cemented Stems: Rationale and Drawbacks

Cemented fixation is the prevailing practice in the investigated cohort, adopted in roughly three-quarters of cases (71.2% cemented without antibiotics and 4.7% with antibiotics), while uncemented stems account for a minority (24.1%). The clinical trade-off remains well known: cementation can mitigate early mechanical problems, such as periprosthetic fractures, but carries perioperative risks, including BCIS, thus requiring vigilant anaesthetic and hemodynamic management in frail patients [8,10,11,14,17,18]. However, in this regard, BCIS is considered rare, especially with the use of modern cements [17,31]. Amin et al. suggests alerting the anaesthesia team at the cementation phase to be ready for resuscitation manoeuvres in case of its occurrence [17]. Moreover, the same authors described the steps used for cement applications [17]. Regarding the risk of periprosthetic fractures, cementless HA has been reported to have an 11-fold increased risk compared to cemented fixation [18].

Antibiotic-loaded cement was used in 6.2% of cemented implants, corresponding to 4.7% of the total cohort, which may reflect cost considerations and selective indications. Although registry data did not allow for a direct assessment of infection risk reduction, several studies have shown that the addition of antibiotics to cement can decrease the incidence of periprosthetic joint infection (PJI), particularly in high-risk individuals [31,32]. In addition, PJI, as reported by De Haan et al., has been associated with a significantly higher one-year mortality, since in PJI 40% of patients died within one year after surgery, compared with 27% in patients without PJI after HA [33].

For this reason, current evidence suggests that targeted use in selected patients, such as those with increased BMI and comorbidities, could be beneficial [32,33,34]. Given the low revision burden in the cohort investigated, especially for septic reasons, a targeted, risk-adapted use appears reasonable, while acknowledging cost and antibiotic stewardship considerations [32,33,34].

### 4.2. Surgical Approach

The lateral approach was the most frequently adopted (52.6%), followed by posterolateral (43.7%) and the anterior (2.9%). Literature reports how surgical approach may influence outcomes and possible complications in hip replacement [13,15,35,36]. Most of the data also derive from total hip replacement, where each surgical approach has advantages and drawbacks [13,15,35,36,37,38,39].

As reported by Morrell et al. in a Cochrane review, in patients undergoing HA for intracapsular hip fractures, the pain and the effect of surgical approach on activities of daily living and pain within four months have uncertain evidence [40]. In addition, there is little to no evidence of a difference in functional status, mortality, and health-related quality of life between the approaches, as well as insufficient evidence to determine whether anterior, lateral, or posterior approaches are a more suitable option for hemiarthroplasty with respect to these outcomes [40].

In the cohort investigated, the approach most used was the direct lateral, likely reflecting its protective effect against dislocation, which in any case is the most frequent cause of failure in this series (46.9%). While concerns about postoperative abductor weakness exist, the reduction in dislocation risk is a compelling argument for its use in elderly patients, where stability is a primary objective [41,42,43]. Indeed, as reported by Jobory et al., the dislocation rate was 7% with the posterior approach compared to 3% with the lateral approach [38]. This could be reduced, as reported by the same authors, with the tendon-to-bone repair of the posterior structures [38]. Barimani et al. underlined the same concept, describing how closing the posterior capsule can decrease the historically high dislocation rate associated with converting a HA to a total hip replacement using the posterolateral approach [35].

The anterior approach is becoming increasingly popular, both for THA and for HA; still, in the analysis performed, it was employed in less than 3% of cases [39,44,45].

In this regard, Nogler et al. described in detail the surgical technique step-by-step employed in DAA for patients over 80 years of age with femoral neck fractures undergoing HA [44]. The authors reported good outcomes with reduced blood loss, less postoperative pain, and a decreased hospital stay compared to the posterior or Hardinge approach [44].

The same was reported by Orth et al., who compared geriatric patients treated with anterior minimal invasive surgery (AMIS) and the lateral approach, describing the first cohort as having less surgery-related strain and recovering faster in the early postoperative phase compared to those treated with the lateral approach after displaced FNFs [45]. Of note is the steep learning curve and surgeon expertise to perform the anterior approach with risk of complications as intraoperative femur fracture [39,44,45]. Indeed, the finding of this investigation supports the preferential use of the lateral approach in this setting, while recognizing that surgeon expertise remains a decisive factor [39,44,45].

Dislocation was the leading cause of failure, underscoring how approach-related stability and soft-tissue management dominate the failure landscape in this cohort. While the analyses presented do not include an approach-stratified survivorship, the descriptive alignment, with the high use of lateral access with dislocation as the primary failure mode, reinforces the centrality of strategies that maximize stability, such as the meticulous repair of posterior structures when applicable [35,38].

### 4.3. Failure Characteristics, Implant Details, and THA

Most cases in the cohort investigated involved bipolar cups and cemented stems. Failures due to mechanical issues such as periprosthetic fracture (13.1%) or aseptic loosening (9.4%) were relatively uncommon, suggesting that current stem designs provide reliable fixation in this population.

The virtual absence of large-diameter heads and ceramic-on-ceramic couples in this series reflects a cautious approach: in elderly patients, the focus is on reducing dislocation and implant breakage rather than on maximizing long-term wear resistance [46,47]. These implant options are reported mainly in the context of total hip replacement rather than hemiarthroplasty, which may explain their limited use in this cohort [48,49,50,51,52].

The distribution of failure mechanisms according to the time from the index surgery in this series supplies relevant clinical insights. Dislocations tended to be clustered in the early postoperative period, emphasizing the need for surgical stability, approach selection, and soft-tissue management [13,15,35,36,46,47,48]. Conversely, aseptic loosening and acetabular erosion appeared later, reflecting both biological and mechanical processes that evolve over time rather than issues involving the index procedure [11,46]. Periprosthetic fractures, as already mentioned, tend to occur all throughout.

Therefore, different preventive strategies should be adopted, specifically a meticulous surgical technique and stability-focused planning to prevent early dislocations, and careful long-term follow-up for the detection of progressive loosening or acetabular wear.

Lastly, worthy to mention and well described in the literature is the use of THA instead of HA for femoral neck fractures [5,6,7,53]. Despite this point not being addressed on purpose, since it should require a separate discussion, as reported by Liu et al., there has been an increase in the use of THA for femoral neck fractures due to surgeons’ and patients’ factors [53]. In this regard, Luo et al. reported more blood loss, longer operation time, and longer hospitalization in THA, with, however, no differences between complications and with the percentage surviving at five years [5]. However, hip function and quality of life were in favor of THA, with no significant differences between groups in hip dislocation rate [5]. Migliorini et al., in a Bayesian network meta-analysis, reported the same, with THA leading to the highest Harris Hip scores and lowest rate of revision surgery compared to bipolar and unipolar hemiarthroplasty [6]. However, they reported bipolar hemiarthroplasty with the lowest dislocation rate when compared with the unipolar or THA [6].

### 4.4. Thromboprophylaxis Management

Venous thromboprophylaxis has been extensively studied in both elective and trauma surgery [54,55,56,57]. In this study, heparin-based prophylaxis was administered to over 93% of patients, demonstrating adherence to the literature [54,55,56,57]. Several other drugs for thromboprophylaxis have been reported with advantages and drawbacks [58,59]. In this regard, for example, Hughes et al. suggested caution in the use of warfarin for thromboprophylaxis for hip and knee arthroplasty due to infection risk [59].

However, in clinical practice, patients on vitamin K antagonists, direct oral anticoagulants, or antiplatelet therapy often require individualized perioperative protocols with a multidisciplinary approach [60]. Unfortunately, registry data cannot capture this complexity.

### 4.5. Mortality

Mortality remains a significant concern after femoral neck fractures in elderly patients, especially in the first year after surgery, but also thereafter [19,61,62]. In this regard, Fakler et al. reported a mortality rate of 13% at 30 days, 25% at one year, and 60% and 80% at five and ten years postoperatively, respectively, in patients 60 years of age or older who underwent bipolar hemiarthroplasty [61]. Literature tends to report mortality rates following proximal femoral fractures from approximately 10% to more than 30% [19,61,62].

In addition, several risk factors have been implicated as predictors of increased mortality risk, including age, the male gender, and a higher ASA score [19,61,62].

The mortality reported in this cohort is in line with the literature [19,61,62], and it highlights the fragility of this cohort since 13.9% died within 90 days and 74.0% died beyond 90 days.

### 4.6. Clinical Relevance

The present study, despite the descriptive nature, underlines that, in elderly patients with femoral neck fractures, surgical priorities in the Emilia Romagna Italian region focus mainly on stability and rapid recovery.

Cemented stems remain the most reliable adopted option in osteoporotic bone. Following the literature, they can ensure immediate fixation and could reduce the risk of early mechanical complications, as periprosthetic fractures [8,10,11,14,16,18]. The lateral approach, adopted in more than half of cases, aligned with the concept reported in the literature that it could minimize the dislocation, which was the leading cause of revision in this descriptive analysis [40,42]. The Kaplan-Meier survival curve with revision of any component defined as the endpoint, either with or without the competing risk of death, showed a good longevity of the implants.

Careful attention to approach selection and soft-tissue management is therefore considered an essential step in surgical planning. Finally, the use of heparin-based thromboprophylaxis was widely adopted.

In an era where the population is aging and the elderly are chronically treated with many medications and have a lot of comorbidities, a multidisciplinary team and approach seem necessary [60]. Overall, secure fixation, stability-oriented surgical strategies, and a multidisciplinary approach represent the cornerstones of care for this fragile yet functionally demanding population [60]. Future prospective studies addressing the clinical parameters along with the registry data will be of paramount importance.

### 4.7. Limitations and Strengths

This study has several limitations. First, the analysis is based on regional registry data, which, although highly reliable in terms of completeness and accuracy, does not include detailed clinical information such as comorbidity indices like ASA scores, perioperative management protocols, functional outcomes, quality of life measures, and therapies. Moreover, collecting data from several orthopedic units, protocols are not always standardized. Data concerning the anticoagulant bridging strategies and surgeon-specific technical details are lacking and could have influenced outcomes. Minor complications, as well as misclassifications of complex revision scenarios, may be underreported due to coding errors, even with standardized procedures. In addition, as the primary focus is HA implants, a comparison with THA was not performed, despite this being a topic highly debated in the literature, with both advantages and drawbacks [5,6,7,53]. Indeed, the recent study by Liu et al. reported an increased trend in the use of THA for femoral neck fractures over the past two decades, due to patients’ and surgeons’ factors affecting treatment decisions [53].

Temporal trends were not analyzed, and in future studies, comparisons and stratification of treatment by year would be worthy to consider changes in time.

Mortality evaluation with the cut-off of 90 days was the timeline subdivision of the RIPO registry. In this regard, literature utilizes the one-year, the five-year, and the ten-year mortality after FNFs. Mortality > 90 days encompasses a very broad category. However, through this subdivision, the perioperative mortality within 90 days is evaluated and underlined.

The observational nature of registry studies precludes definitive causal inference; therefore, associations cannot be concluded, and unmeasured confounding cannot be ruled out.

Lastly, univariate and multivariate analyses of single risk factors, especially concerning dislocation, which is the main cause of failure, were not performed.

Despite these limitations, the huge sample size, prospective data collection, and high completeness of follow-up lend robustness to the results and give insight for surgeons’ options in everyday clinical practice.

Future high-quality studies, as prospective studies, will be necessary to determine the optimal solutions in this frail elderly population.

## 5. Conclusions

This registry-based study, encompassing more than 43,000 hemiarthroplasties performed for femoral neck fractures in patients aged 75 years and older, provides insight to guide surgical decision-making in this fragile population.

Cemented stems were the predominant fixation method, while the lateral approach was the most adopted surgical access, consistent with minimizing instability. Revision rates were low, but dislocation accounted for nearly half of all failures, underscoring the importance of stability-focused strategies in surgical planning. Mechanical failures such as fracture, loosening, or wear were less common. Thus, the priorities in treating elderly patients with femoral neck fractures remain secure fixation, surgical approaches that reduce dislocation risk, and multidisciplinary perioperative protocols to mitigate systemic complications. These findings reinforce current practice patterns and provide insights for optimizing outcomes in this growing and fragile patient population.

## Figures and Tables

**Table 1:** Demographic details of patients (RIPO database). Percentages are calculated on available data; missing data are reported separately.

Demographic Details	n (%)
**Sex**	
Female	32,195 (73.7)
Male	11,462 (26.3)
**Age (years)**	
Median (IQR)	85.0 (81.0–89.0)
Mean (SD)	85.5 (5.5)
**BMI**	
Underweight	1684 (5.4)
Normal weight	16,865 (53.6)
Overweight	10,838 (34.5)
Obese	2069 (6.6)
Unknown	12,201

n: number; SD: standard deviation; IQR: interquartile range; BMI: body mass index (defined according to the WHO as underweight BMI < 18.5, overweight BMI ≥ 25, and obese BMI ≥ 30) [30].

**Table 2:** Perioperative details of patients (RIPO database). Percentages are calculated on available data; missing data are reported separately.

Perioperative Details	n (%)
**Mortality (cut-off 90 days from the index surgery)**	
Death after 90 days from the index surgery	32,292 (74.0)
Death within 90 days from the index surgery	6086 (13.9)
Alive	5279 (12.1)
**Heparins**	
Yes	40,751 (93.4)
No	2865 (6.6)
Unknown	41
**Blood Transfusions**	
Yes	21,957 (62.0)
No	13,431 (38.0)
Unknown	8269

n: number.

**Table 3:** Implant details (RIPO database). Percentages are calculated on available data; missing data are reported separately.

Implant Details	n (%)
**Fixation of stem**	
Cemented, no antibiotics	31,012 (71.2)
Cemented, with antibiotics	2050 (4.7)
Uncemented, coated	6511 (14.9)
Uncemented, uncoated	3999 (9.2)
Unknown	85
**Neck**	
Fixed	40,592 (93.0)
Modular	3062 (7.0)
Unknown	3
**Monoblock versus Bipolar cups**	
Bipolar to assemble in the OR	42,242 (96.8)
Bipolar already assembled	950 (2.2)
Monoblock head	461 (1.1)
Unknown	4
**Diameter of the femoral head (mm)**	
≤28	41,552 (99.1)
32	395 (0.9)
≥36	1 (0.0)
Unknown	1507
**Material of the femoral head**	
Metallics	41,245 (98.5)
Ceramics	592 (1.4)
Others	46 (0.1)
Unknown	1.774
**Surgical approach**	
Lateral	22,864 (52.6)
Posterolateral	18,975 (43.7)
Anterior	1256 (2.9)
Other/Unknown	562 (1.3)

n: number; OR: operating room; mm: millimeters.

**Table 4:** Causes of failures leading to revision surgery (n = 853 revisions). Dislocation represented nearly half of all recorded failures, followed by periprosthetic fracture, acetabular wear, and aseptic and septic loosening.

Cause of Failure	n	% of Total Failures	Incidence Rate (%)
Dislocation	400	46.9%	0.9
Periprosthetic fracture	112	13.1%	0.3
Acetabular wear	81	9.5%	0.2
Aseptic stem loosening	80	9.4%	0.2
Septic loosening	67	7.9%	0.2
Unknown/not specified	65	7.6%	0.1
Others	33	3.9%	0.1
Primary instability	15	1.6%	0.0

n: number. “Unknown” refers to reasons for revision not specified, while “Others” refers to other causes, such as implant breakage, pain not related to the above, etc.

**Table 5:** Type of revision procedures performed (n = 853 revisions). Conversion to total hip arthroplasty accounted for most revision procedures.

Revision Procedures	n	% of Revisions	Incidence Rate (%)
Conversion to THA	454	53.2	1.0
HA revision	283	33.2	0.6
Complete removal of prosthetic material	116	13.6	0.3

n: number; THA: total hip arthroplasty; HA: hemiarthroplasty.

**Table 6:** Subtypes of the revision procedures performed (N = 853 revisions).

Subtypes and Details of the Revision Procedures	n	% of Revisions	Incidence Rate (%)
Acetabular component positioning	335	39.3	0.8
Revision of all HA components	214	25.1	0.5
Complete removal of prosthetic material	116	13.6	0.3
Isolated cup revision	106	12.4	0.2
Isolated HA stem revision	52	6.1	0.1
Isolated HA head revision	26	3.0	0.1
Isolated HA neck revision	4	0.5	0.0

n: number; THA: total hip arthroplasty; HA: hemiarthroplasty.

## Data Availability

The data that support the findings of this study are available from the corresponding Author upon reasonable request. Some data are publicly available at RIPO (http://ripo.cineca.it/authzssl/index.htm (accessed on 17 February 2025)).

## Abbreviations

FNFs	femoral neck fractures
RIPO	Registro dell’Implantologia Protesica Ortopedica Emilia Romagna
HA	hemiarthroplasty
PMMA	polymethylmethacrylate
THA	total hip arthroplasty
RIA	Registro Italiano Artroprotesi
SD	standard deviation
mm	millimeters
n	number
IQR	interquartile range
BMI	body mass index
OR	operating room
BCIS	bone cement implantation syndrome
PJI	periprosthetic joint infection
AMIS	anterior minimal invasive surgery
DAA	direct anterior approach
ASA	American Society of Anesthesiologists
